# Accuracy and usefulness of BMI measures based on self-reported weight and height: findings from the NHANES & NHIS 2001-2006

**DOI:** 10.1186/1471-2458-9-421

**Published:** 2009-11-19

**Authors:** Manfred Stommel, Charlotte A Schoenborn

**Affiliations:** 1 College of Nursing, Michigan State University, W-149 Owen Graduate Center, East Lansing, Michigan 48825-1109, USA; 2 CDC/National Center for Health Statistics, 3311 Toledo Rd. Room 2331, Hyattsville, MD 20782, USA

## Abstract

**Background:**

The Body Mass Index (BMI) based on self-reported height and weight ("self-reported BMI") in epidemiologic studies is subject to measurement error. However, because of the ease and efficiency in gathering height and weight information through interviews, it remains important to assess the extent of error present in self-reported BMI measures and to explore possible adjustment factors as well as valid uses of such self-reported measures.

**Methods:**

Using the combined 2001-2006 data from the continuous National Health and Nutrition Examination Survey, discrepancies between BMI measures based on self-reported and physical height and weight measures are estimated and socio-demographic predictors of such discrepancies are identified. Employing adjustments derived from the socio-demographic predictors, the self-reported measures of height and weight in the 2001-2006 National Health Interview Survey are used for population estimates of overweight & obesity as well as the prediction of health risks associated with large BMI values. The analysis relies on two-way frequency tables as well as linear and logistic regression models. All point and variance estimates take into account the complex survey design of the studies involved.

**Results:**

Self-reported BMI values tend to overestimate measured BMI values at the low end of the BMI scale (< 22) and underestimate BMI values at the high end, particularly at values > 28. The discrepancies also vary systematically with age (younger and older respondents underestimate their BMI more than respondents aged 42-55), gender and the ethnic/racial background of the respondents. BMI scores, adjusted for socio-demographic characteristics of the respondents, tend to narrow, but do not eliminate misclassification of obese people as merely overweight, but health risk estimates associated with variations in BMI values are virtually the same, whether based on self-report or measured BMI values.

**Conclusion:**

BMI values based on self-reported height and weight, if corrected for biases associated with socio-demographic characteristics of the survey respondents, can be used to estimate health risks associated with variations in BMI, particularly when using parametric prediction models.

## Background

The use of the Body Mass Index (BMI) based on self-reported height and weight in epidemiologic studies remains controversial, both because it is an imperfect measure of a person's percentage of body fat [1] and because self-reported height and weight are subject to substantial measurement error [2]. However, BMI measures calculated from data obtained through interviews continue to be used as a common tool because of the relative ease and efficiency in gathering such information. Thus, it remains important to assess the extent of error present in BMI measures that are based on self-reported height and weight (self-reported BMI) compared with BMI measures based on physical measurement of height and weight (measured BMI) and to understand the limitations of using the self-reported measures.

A review of the literature reveals both random and systematic errors ("bias") in the use of self-reported measures, but there remains some controversy over the magnitude of such errors. In particular, researchers have shown that over-reporting of height is more common among older survey respondents [3,4]. In addition, it has often been reported that men are more likely than women to overestimate their height, while women, particularly young women, are more likely to underestimate their weight [3,5]. Some researchers have also found systematic variations in the BMI based on the racial or ethnic background of interview respondents. For instance, Mexican Americans and, in particular, Mexican American women tend to underestimate their weight, resulting in underestimates of the prevalence of obesity in this group [6]. In a study of Mexican citizens, others also found that BMI values calculated from self-reported height and weight *under*estimated BMI values based on measured height and weight --- a tendency that increased with age [7]. However in one study [8], it was reported that Mexican American adults tend to underestimate their weight *less *than non-Hispanic white adults. A similar pattern also seems to hold for non-Hispanic African American adults, for whom BMI values computed from self-reported height and weight seem to produce smaller underestimates of their measured BMIs than among non-Hispanic whites [6,8]. Conversely, compared to non-Hispanic African American and Hispanic women, non-Hispanic white women are *more *prone to underestimate their weight [4], which may reflect different sensitivities about being overweight [9]. Marital status and income also appear to influence a survey participants' responses to height and weight questions, with single/never married as well as divorced persons more likely to underreport weight, and high income respondents reporting their weight and height more accurately [8,10]. Finally, there is strong evidence that the actual BMI itself is a predictor of the error in BMI measures based on self-reported height and weight, with underestimates of BMI becoming larger for respondents with higher-end weight and BMI values [11-13].

While developing correction factors that could be applied to self-reported data to eliminate, or at least lessen, the biases in BMIs associated with self-reported height and weight is an attractive idea [14], the few attempts that have been made [15,16] are discouraging. Use of additional variables beyond self-reported height and weight provides only small improvements in the prediction of measured BMI scores and does not appear to eliminate biases associated with the size of the measured BMI [16].

Beyond the question of possible errors and biases in BMI measures based on self-reported height and weight, there is also the question of the purpose for which self-reported BMI measures are employed. For instance, biases in self-report may have a great impact on our ability to classify people as "overweight" or "obese", but may have a smaller impact on the use of a continuous BMI measure to estimate risks associated with body mass. In much of the literature, researchers employ the BMI categories based on the 1998 National Heart, Lung, and Blood Institute (NHLBI) Guidelines, which use the following groupings: BMI < 18.5 ("underweight"), 18.5 < 25 ("normal weight"), 25 < 30 ("overweight"), 30 < 35 ("low obesity"), 35 < 40 ("medium obesity"), and ≥ 40 ("extreme obesity"). Even though a comparison of BMI categories based on measured and self-reported height and weight may reveal substantial misclassifications, it is unclear whether such misclassification is based on small or large deviations between self-reported and measured BMI values. If the errors tend to be small, the use of continuous BMI measures based on self-reported height and weight may result in fairly accurate estimates of the morbidity and mortality risks associated with the BMI, even though relatively large misclassifications occur at the margins with the use of broadly defined BMI categories. This study is designed to provide estimates of the extent to which survey respondents are misclassified with respect to their body mass categories if BMI estimates computed from self-reported weight and height are used. We will also provide estimates of the overall measurement errors involved in using self-report measures and examine the predictors reviewed, while adding an indicator for current pregnancy, on the assumption that temporary weight gain may lead to under-reporting of weight. Since it has been well established that the risk of diabetes rises with higher BMI values [17-19], we will analyze to what extent the association between the BMI and the risk of diabetes differs, depending on whether we use physical measures to calculate BMI or BMI values computed from self-reported height and weight. Finally, an attempt is made to adjust BMI values based on self-reported height and weight to improve their accuracy in classifying respondents as "overweight" or "obese."

## Methods

The following analysis is based on the combined 2001-2006 data from the continuous National Health and Nutrition Examination Survey (NHANES) conducted by the National Center for Health Statistics (NCHS). The NHANES surveys are multi-stage probability samples of the non-institutionalized U.S. civilian population. Both physical measurement and self-reported height and weight information are collected in the NHANES for the same individuals, with the questionnaire preceding the physical examinations. Physical examinations take place in mobile examination centers and body measurements are based on standard protocols [20]. The overall unweighted NHANES response rates for the interview samples of the three continuous two-year surveys were 84% (2001-2002), 79% (2003-2004), and 80.5% (2005-2006) [21].

The combined 2001-2006 NHANES sample includes 17,176 adults. Of these, 15,662 (91.2%) had BMI values based on physical measures of height and weight, and 16,579 (96.5%) had BMI values based on self-reported height and weight. For 15,161 (88.3%) adult NHANES participants, both self-reported and measured BMI indicators were available for analysis. Among these, 15,155 adults also provided responses to the question: "Have you EVER been told by a doctor or health professional that you have diabetes or sugar diabetes?" Response rates for the height and weight information were lower among older respondents (particular those over 70 years of age) and among Hispanics compared to whites and African Americans, largely due to lower response rates for the measured height and weight data. Gender differences were generally small, with men having slightly better response rates.

For comparison estimates, self-reported data from the 2001-2006 National Health Interview Survey (NHIS) Sample Adult components were also analyzed. The NHIS is a household, multi-stage probability sample survey conducted annually by NCHS and fielded by the U.S. Census Bureau for the Centers for Disease Control and Prevention's National Center for Health Statistics [22]. The sample size for the combined 2001-2006 NHIS Sample Adult interview was 182,251 adults. Of these, 95.3% had BMI values based on their self-reported height and weight. The average annual response rate for the 2001-2006 NHIS household surveys was 88%; the final response rate for the Sample Adult component (for which self-response is required) was 72%.

The weight history section of the NHANES interview contained the following two questions: (1) "How tall are you without shoes?" This question could be answered by the respondent in terms of feet and inches or meters and centimeters; and (2) "How much do you weigh without clothes or shoes?" This question could be answered in terms of pounds or kilograms. The parallel NHIS questions were: (1) "How tall are you without shoes?" and (2) "How much do you weigh without shoes?"

Both the NHANES and NHIS surveys are subject to the CDC/NCHS Ethics Review Board (ERB) to ensure that appropriate human subjects protections are provided, in compliance with 45 CFR part 46. All statistical analyses were carried out with STATA 10.1 software [23], using the "svy" command to incorporate information on the appropriate weights, primary sampling units and strata for correct variance estimation. This analysis is limited to the adult (18 years and over) samples in both surveys. Estimates are age-standardized based on the 2000 U.S. Census results, using 10-year age intervals.

## Results

The results in Table 1 show a substantial amount of misclassification of the BMI based on self-reported height and weight (self-reported BMI) compared with the BMI based on measured height and weight (measured BMI). More than 43% of respondents classified as "underweight" and 16% of respondents classified as "overweight" based on their measured BMI were classified as "normal weight" using the self-reported BMI. In addition, 19% of respondents classified as "obese" using measured BMI were misclassified as "overweight" using self-reported BMI. The general trend is for classification errors to be larger in the extreme (over- or underweight) categories. Sensitivity values (the proportions of overweight or obese persons according to physical measurement, who are classified as overweight or obese according to their self-reported measures) are 91.4% for overweight or more and 83.3% for the obesity classification. The corresponding positive predictive values (the proportion of self-reported 'overweight' or 'obese' persons who actually are overweight or obese based on measured height and weight) are 95.8% and 93.9%, respectively. However, a closer look at the misclassifications reveals that the majority of the misclassified cases have BMI values within an interval of just one unit from the category boundary in question. For example, while 43.5% of adults classified as "underweight" based on their measured BMI were classified as "normal weight" based on self report, about three-fourths of these individuals (32.1%) had self-reported BMI values at the lower end of "normal" category --- with a self-reported BMI value between 18.5 and 19.5. Similarly, while 16.0% of overweight adults were misclassified as "normal weight" using self-reported data, nearly two-thirds of these overweight adults (10%) had a self-reported BMI values between 24 and 25; likewise, 19% of obese persons were misclassified as "overweight" using self-reported BMI, but more than half of these adults (10.6%) had a self-reported BMI value between 29 and 30. Finally, among extremely obese individuals whose self-reported BMI fell below 40, 9.7% actually had a self-reported BMI between 39 and 40. Generally, deviations of BMI values based on self-reported height and weight from BMI values based on measured height and weight are moderate: an estimated 56% have self-reported BMI values within a one-unit interval of their measured BMI, and 81.5% have self-reported BMI values within two units of their measured BMI (not shown). More extreme deviations tend to occur at the very top (underestimates) and bottom (overestimates) of the BMI distribution.

**Table 1:** Cross-Classification of Measured BMI and Self-Reported BMI for Standard BMI Categories*

	Measured BMI:				
**Self Reported BMI**:	***Under-weight*** ***< 18.5***	***Normal Weight*** ***18.5 < 25***	***Over-*** ***weight*** ***25 < 30***	***Obese*** ***30 < 40***	***Extreme Obesity*** ***40+***

*< 18.5*	56.5%	1.5%	0.1%	0.0%	0.0%

*18.5 < 25*	43.5%	90.4%	16.0%	0.8%	0.0%

*25 < 30*	0.0%	8.0%	78.9%	19.0%	0.3%

*30 < 40*	0.0%	0.1%	5.1%	78.5%	27.8%

*40+*		0.0%	0.0%	1.6%	71.9%

N = 15161; Population Size: 193,275,651					

* Measured BMI was calculated using physical measurements of height and weight;

Self-reported BMI was calculated using self-reported height and weight.

Source: National Health and Nutrition Examination Survey, 2001-2006. National Center for Health Statistics, CDC

Table 2 displays arithmetic means for self-reported (SR) and measured (M) heights, weights and BMIs, as well as the discrepancies (DIS) between the self-reported and measured variables. In addition to information about the standard errors, standardized effect sizes for the discrepancy scores are added. As the rightmost column shows, the overall population mean for self-reported heights represents a modest overestimate of one centimeter, while self-reported weight represents an underestimate of measured weight by 3/4 of a kilogram (1.6 pounds). The net result is that the mean population estimate of the BMI, based on the self-reported heights and weights, is lower than the mean estimate of the measured BMI by 0.59 units. The effect sizes of the height and weight discrepancies for the total population suggest that height overestimates contribute more to the total population BMI discrepancies than weight underestimates. However, the discrepancies in each BMI class hint at linear trends such that overestimates of self-reported height become larger with larger measured BMI categories. The weight discrepancies indicate overestimates of self-reported weight in lower BMI categories and underestimates of self-reported weight in the higher BMI categories. The net result is a linear trend towards declining self-reported BMI values relative to measured BMI values. A linear regression model predicting the discrepancy between self-reported and measured BMI values (BMI_DIS_) based on the measured BMI (BMI_M_) values leads to the following estimates: BMI_DIS _= 2.283 - 0.102 BMI_M_, with standard errors of 0.134 for the intercept and 0.005 for the regression coefficients. Using this equation, self-reported BMI values would be unbiased estimates (i.e., BMI_DIS _= 0) of actual (measured) BMI for persons with a BMI of 22.4 (2.283/0.102), but would overstate actual BMI at lower BMI values and understate it at higher BMI values. Furthermore, the effect sizes for the height and weight discrepancies in the underweight category suggest that weight overestimates play a large role in the BMI overestimates in this group. By comparison, the effect sizes among the obese indicate that both height overestimates and weight underestimates contribute in equal measure to BMI underestimates.

**Table 2:** Means of self-reported, measured, and discrepancy scores of height, weight and BMI values by BMI categories based on measured height and weight

	**BMI Categories based on measured height and weight**:					
**SR^a^, M^b^, DIS^c^** **Measures**:	***Under-weight*** ***< 18.5***	***Normal Weight*** ***18.5 < 25***	***Over-weight*** ***25 < 30***	***Obese*** ***30 < 40***	***Extreme Obesity*** ***40+***	***Total Population***

Height-SR^a ^{cm}						
*Mean* *(SE^d^)*	168.2 (0.71)	169.3 (0.21)	171.4 (0.18)	170.1 (0.22)	168.7 (0.48)	170.2 (0.12)

Height-M^b ^{cm}						
*Mean* *(SE^d^)*	167.7 (0.69)	168.7 (0.22)	170.3 (0.17)	168.9 (0.22)	167.4 (0.47)	169.2 (0.11)

Height-DIS^c ^{cm}						
*Mean* *(SE^d^)* *[ES^e^]*	0.50 (0.13) [0.19]	0.58 (0.05) [0.20]	1.11 (0.06) [0.38]	1.16 (0.06) [0.42]	1.24 (0.13) [0.43]	0.95 (0.04) [0.33]

Weight-SR^a ^{kg}						
*Mean* *(SE^d^)*	51.8 (0.48)	64.5 (0.21)	79.2 (0.17)	94.2 (0.33)	122.9 (0.86)	80.1 (0.33)

Weight-M^b ^{kg}						
*Mean* *(SE^d^)*	49.7 (0.46)	64.0 (0.19)	79.8 (0.16)	96.2 (0.29)	127.2 (0.86)	80.9 (0.33)

Weight-DIS^c ^{kg}						
*Mean* *(SE^d^)* *[ES^e^]*	2.14 (0.16) [0.75]	0.53 (0.06) [0.17]	-0.60 (0.07) [-0.17]	-2.02 (0.10) [-0.42]	-4.31 (0.40) [-0.49]	-0.75 (0.05) [-0.17]

BMI-SR^a ^{kg/m^2^}						
*Mean* *(SE^d^)*	18.2 (0.07)	22.4 (0.04)	26.8 (0.04)	32.5 (0.07)	43.1 (0.25)	27.6 (0.10)

BMI-M^b ^{kg/m^2^}						
*Mean* *(SE^d^)*	17.6 (0.05)	22.4 (0.03)	27.4 (0.02)	33.6 (0.06)	45.2 (0.22)	28.2 (0.11)

BMI-DIS^c ^{kg/m^2^}						
*Mean* *(SE^d^)* *[ES^e^]*	0.64 (0.07) [0.59]	0.02 (0.03) [0.02]	-0.56 (0.03) [-0.37]	-1.16 (0.04) [-0.58]	-2.12 (0.16) [-0.62]	-0.59 (0.02) [-0.31]

N = 15,161; Population Size: 193,275,651						

^a ^SR = Self-reported (SR) in interview, ^b ^Physical measure (M), ^c ^Discrepancy between Self-reported and physical measures (DIS): positive values represent overestimates, negative values represent underestimates, ^d ^*SE *= Standard Error, ^e ^*ES *= Effect Size

Source: National Health and Nutrition Examination Survey, 2001-2006. National Center for Health Statistics, CDC

Three multivariate linear regressions were conducted to predict variations in the discrepancy variables, i.e., the over- or underestimates of height (R^2 ^= 0.061, p ≤ 0.001), weight (R^2 ^= 0.070, p ≤ 0.001) and BMI (R^2 ^= 0.045, p ≤ 0.001). All three models included the same set of predictors: gender, age, race/ethnicity (non-Hispanic white, Mexican American, other Hispanic, non-Hispanic black, other), marital status (married, widowed, divorced, separated, single/never married, living with a partner), education (less than high school, finished high school/GED, some college or more), pregnancy status and household income (less than $20,000 vs. $20,000 or more). The first model showed that overestimation of *height *was greater among non-Hispanic blacks (+0.15, p < 0.05) and Hispanics other than Mexican Americans, (+0.43, p < 0.03), than among all other racial/ethnic groups. In the second model, overestimation of self-reported *weight *occurred among widowed respondents (+.40, p < 0.02) compared with married individuals, weight was underestimated among college educated individuals (-0.51, p < 0.01) compared with individuals with less than a high school education, while pregnant women underestimated their weight, on average, more than 5 kg (-5.43, p < 0.01). The net effect of race/ethnicity, marital status, education, household income and pregnancy status on over- or underestimating the BMI remained small: only college-educated individuals and pregnant women had self-reported BMI values that were significant underestimates (-.13, p < 0.01 and -2.07, p < 0.01 respectively) of their measured BMIs.

**Figure 1:**
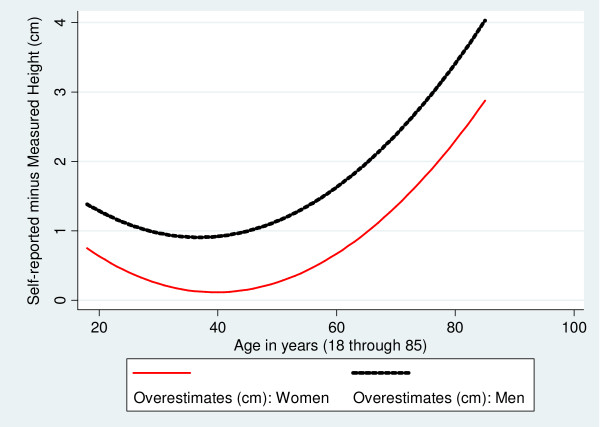
**Associations of Gender and Age with Discrepancies between Self-reported and Measured Height: Fitted Values**.

**Figure 2:**
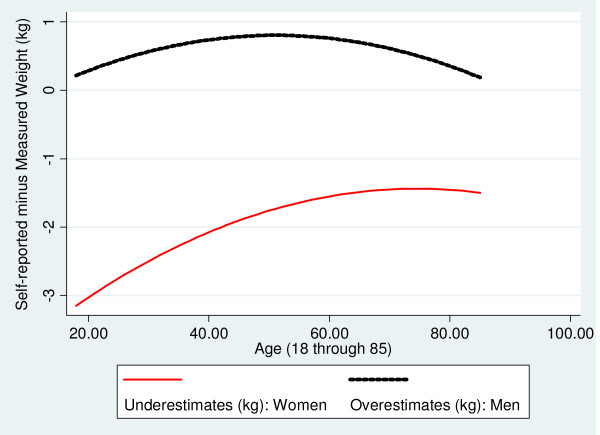
**Associations of Gender and Age with Discrepancies between Self-reported and Measured Weight: Fitted Values**.

**Figure 3:**
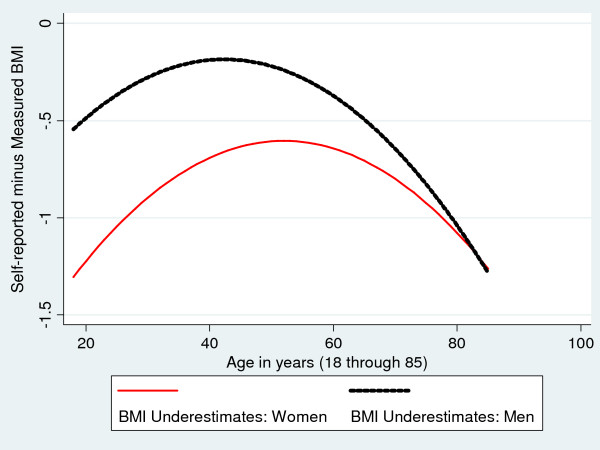
**Associations of Gender and Age with Discrepancies between Self-reported and Measured BMI: Fitted Values**.

Figures 1, 2, 3 are based on the same regression models and illustrate how age and gender are associated with the discrepancies between measured and self-reported height, weight and BMI. Both men and women overstate their height, particularly at older ages, although the extent of over-reporting is greater for men than for women, p < 0.01 (Figure 1). Men also tend to overstate their weight, although by relatively small amounts (on average, less than 1 kg) (Figure 2). In contrast, women *understate *their measured weight with the greatest understatement (on average, more than 3 kg) found among young women (Figure 2). The net effect on the BMI of these patterns in self-reporting of height and weight is depicted in Figure 3. Although self-reported BMI understates measured BMI for both men and women across all age groups, the discrepancy is significantly greater (p < 0.001) for women except at the very oldest ages. For both genders, the most accurate BMI values based on self-reported height and weight are obtained between the ages 42 and 55, with larger underestimates of measured BMI among the youngest and oldest individuals.

Given the pattern that self-reported BMI values underestimate "true" BMI values, except at very low levels of BMI, and given that the discrepancies between self-reported and measured BMI vary systematically with height, weight, age, gender, pregnancy status, marital status, income, and, to a lesser extent, with race and ethnicity, we used these variables to predict measured BMI scores (Table 3). The results from this regression model show that self-reported height and weight, in conjunction with a few demographic characteristics, account for more than 92% of the variation in measured BMI scores. (Note: We used a polynomial regression approach for height and weight and age, eliminating higher power terms, if they showed no significant effect on the dependent variable [24].) The predicted BMI scores from this model represent "adjusted" BMI scores, which take account of all the predictor variables in the equation. While the mean discrepancy between the adjusted and measured BMI is close to zero, since it is a residual score, these discrepancies continue to show a systematic, though smaller, bias in relation to the measured BMI. The simple linear regression model using the measured BMI values as predictors of the adjusted discrepancy scores yields the following results: BMI-(Adjusted) Discrepancy = 2.204 - 0.078 BMI, with standard errors of 0.109 for the intercept and 0.004 for the coefficient. Based on this equation, the actual BMI value at which the adjusted self-reported BMI scores are unbiased is 28.3 (2.204/0.078). Adjusted self-reported BMI values in the range below 28.3 are overstated and are understated in the range above 28.3. However, the biases are not large; at an actual BMI of 18.5, the average overestimate is 0.78 BMI units, while the average underestimate at a BMI of 40 is 0.91 units.

**Table 3:** Regression of BMI, based on Measured Height and Weight, on Self-reported Height, Weight & Demographic Predictors (NHANES 2001-2006)

	*Regression* *Coefficient*	*Standard* *Error*	*P-value*
Height-SR^a ^[cm]	0.268	0.123	0.00

(Height-SR^a^)^2 ^[cm^2^]	-0.002	0.0003	0.00

Weight-SR^a ^[kg]	0.476	0.008	0.00

(Weight-SR^a^)^2 ^[kg^2^]	-0.001	0.0001	0.00

Gender (1 = f, 0 = m)	1.261	0.059	0.00

Age (in years)	-0.032	0.007	0.00

Age^2^	0.0004	0.00007	0.00

Pregnant (1 = y, 0 = n)	2.037	0.135	0.00

Race/Ethnicity^b^			

_Mexican American	0.266	0.065	0.00

_African American	0.064	0.045	0.16

_Other Minorities	0.334	0.081	0.00

Marital Status^c^			

_Widowed	-0.147	0.077	0.06

_Divorced/Separated	0.020	0.063	0.75

_Never Married	0.184	0.05	0.00

_Living with Partner	0.128	0.078	0.11

Household income^d^			

_≥$20,000	-0.132	0.050	0.01

^a ^SR = Self-report measure from interview

Reference categories: ^b ^Non-Hispanic White, ^c ^Married, ^d ^HH Income < $20,000

Subpopulation of observations: 15,159, Estimated Population Size: 193,189,727, Design df = 45; F(17, 29) = 8120.48; Prob > F = 0.000; R-squared = 0.922

Classification of individuals as overweight or obese is improved by employing the adjusted BMI score, predicted from the regression model shown in Table 3. The adjusted BMI score improves the sensitivity of being classified as overweight or more (BMI 25+) to 94.6% (from 91.4%) and the sensitivity of being classified as obese (BMI of 30+) to 91.5% (from 83.3%). Population estimates are also improved, as shown in Table 4. The three NHANES estimates of the percentages of overweight and obese individuals in the population show that the adjusted self-reported measure mirrors the BMI categories based on measured height and weight more closely than the unadjusted self-reported measure. The NHIS, which exclusively relies on interview data, yields a very low estimate of the percentage of obese U.S. residents, even when compared to the interview data from the NHANES. However, after applying the NHANES prediction model to the NHIS to adjust for systematic biases in self-reporting height and weight, population estimates of overweight and obesity among U.S. adults using data from the NHIS more closely approximate the NHANES (measured) estimates, although significant differences remain for the obesity category.

**Table 4:** Estimates of U.S. Civilian Resident Population by BMI Categories using Physical Measures, Self-Reports, and Adjusted Self-Reports

	NHANES 2001-2006:		
	***BMI based on Physical Measures***	***BMI based on Self-reported Measures***	***BMI based on Adjusted Self-reported Measures***

BMI Categories:	% (S.E.^1^)	% (S.E.)	% (S.E.)

*Under-weight* *< 18.5*	2.0 (0.15)	1.6 (0.14)	1.8 (0.14)

*Normal Weight* *18.5 < 25*	32.8 (0.61)	36.1 (0.57)	32.1 (0.59)

*Over-weight* *25 < 30*	33.6 (0.59)	34.2 (0.58)	33.5 (0.58)

*Obesity* *30+*	31.6 (0.75)	28.1 (0.76)	32.7 (0.75)

	**NHIS 2001-2006:**		

		***BMI based on Self-reported Measures***	***BMI based on Adjusted Self-reported Measures***

BMI Categories:		% (S.E.)	% (S.E.)

*Under-weight* *< 18.5*		2.0 (0.05)	2.1 (0.05)

*Normal Weight* *18.5 < 25*		38.7 (0.17)	34.8 (0.17)

*Over-weight* *25 < 30*		35.3 (0.15)	34.8 (0.14)

*Obesity* *30+*		24.0 (0.15)	28.4 (0.15)

^1 ^S.E. = Standard Error

Table 5 shows selected results from five logistic regression models (three based on the 2001-2006 NHANES and two based on the 2001-2006 NHIS) used to predict the odds of having ever been diagnosed with diabetes, based on the BMI measures. All five multivariate logistic regression models include an identical set of covariates: gender, age, education, race/ethnicity and household income. From the odds ratios associated with the BMI measures (both in linear and quadratic form), it is apparent that the adjusted self-reported measures deliver risk estimates that are close to those obtained on the basis of the measured BMI scores. In particular, the estimates obtained from the adjusted NHIS data are almost identical in size to those for the BMI scores based on measured height and weight in the NHANES.

**Table 5:** Odds Ratios predicting prevalence of Diabetes on the basis of measured, self-reported and adjusted self-reported (SR) BMI scores with a standard set of covariates^a^

	NHANES 2001-2006:		
	***Odds Ratio***	***Standard Error***	***P-value***

Model 1:			

Measured BMI	1.209	0.038	0.00

(Measured BMI)^2^	0.999	0.0004	0.00

Model 2:			

Self reported BMI	1.242	0.035	0.00

(Self reported BMI)^2^	0.998	0.0004	0.00

Model 3:			

Adjusted SR BMI	1.198	0.039	0.00

(Adjusted SR BMI)^2^	0.999	0.0005	0.00

	**NHIS 2001-2006:**		

	***Odds Ratio***	***Standard Error***	***P-value***

Model 2:			

Self reported BMI	1.268	0.017	0.00

(Self reported BMI)^2^	0.998	0.0002	0.00

Model 3:			

Adjusted SR BMI	1.207	0.020	0.00

(Adjusted SR BMI)^2^	0.999	0.0002	0.00

^a ^Adjusted odds ratios are based on multivariate logistic regression models with the following set of covariates in the models: gender, age, education, race/ethnicity and household income

Finally, in order to provide independent evidence of the applicability of the adjustments of BMI scores based on self-rated height and weight, we used the regression weights from Table 3 and created an adjusted BMI score using the 1999-2000 NHANES sample of adult respondents (N = 5448). As with the 2001-2006 NHANES data, the adjusted BMI score from the prediction model improves the sensitivity of being classified as overweight or more (BMI 25+) to 94.2% (from 91.2%) and the sensitivity of being classified as obese (BMI of 30+) to 90.9% (from 83.9%).

## Discussion

Our comparison of BMI values based on self-reports and physical measurements confirms several of the basic findings in the literature: While self-report and measured height and weight tend to be highly correlated, there remain deviations of the self-reported BMI values from measured BMI values, particularly at the high and low ends of the BMI scale, which are large enough to result in substantial misclassifications of either underweight or obese people (see Table 1). This is not surprising, given the social sensitivity generally associated with body images [10,11,14]. However, the consensus in the literature -- that the use of self-reported BMI measures leads to substantial underestimates of the population proportion who are overweight and obese -- needs some refinement. For instance, as we have shown, underestimates of the actual BMI based on self-reported height and weight are generally less severe among adults in the age range of 35 to 55, and the bias towards greater underreporting among women than men tends to disappear with older age. More importantly, it is possible to adjust self-reported BMI scores, based on a few, easily gathered demographic characteristics (gender, age, race/ethnicity, marital and pregnancy status, and household income), and thus to obtain a closer approximation to the measured BMI. Of these, age and gender as well as pregnancy status appear to contribute most towards adjusting BMI scores. This is not surprising, as older persons tend to over-report height, probably because they recall their taller selves of yesterday [3], and younger women in particular tend to under-report weight, probably due to a social desirability response [4]. That pregnant women under-report their weight, but not their height, should also not be surprising. While the remaining systematic biases are consistent with those reported in the literature [15,16], overall estimates of the population prevalence of overweight and obesity are nonetheless improved substantially by using the adjusted measures.

What is more, it appears that the estimates of health risks associated with the adjusted self-reported scores of the BMI are very similar to the risk estimates based on the measured BMI, if parametric predictors are used. This should not be surprising, given that more than 80% of the deviations of self-reported BMI from measured BMI do not exceed values within ± 2 BMI units.

While differences in the population estimates of the percentages of overweight U.S. residents are within the margins of sampling errors, the adjusted estimate of the percentage of obese U.S. residents from the NHIS remains about 3 percentage points below the estimate from the NHANES based on measured height and weight. This failure to completely adjust for these differences may well be due to a major limitation in the self-reported measures of the NHANES: respondents knew when they answered these questions that they would undergo a physical examination and might have anticipated that they would be weighed and their height be measured. This may be one reason why the NHIS estimate of obese adults, using the *unadjusted *BMI based on self-reported height and weight, is more than four percentage points lower than the parallel NHANES estimate and more than six percentage points below the NHANES estimate based on measured height and weight. In short, the lack of anticipation of an actual measurement may have led to a less "realistic" self-reporting in the NHIS. Clearly, what is needed for even better BMI adjustments are comparisons of self-reported and measured height and weight from population-based surveys, in which respondents would not anticipate being measured, when asked to report on their height and weight.

Beyond the question of accuracy in measurement, the use of the BMI as predictor of various health risks also depends on the health risks at issue. For instance, some researchers [25] have shown that the BMI is just as good a predictor of the incidence of diabetes as a waist-hip ratio or waist circumference. By contrast, others [26] reported that the BMI performed somewhat less well than waist circumference as a measure of risk for cardiovascular diseases. Still, given the costs of data collection and the ability to reduce, even though not eliminate, systematic biases through adjustments using a few demographic variables, BMI values based on self-reported height and weight remain an important tool for population-based estimates of the health risks involved in obesity.

BMI values based on self-reported height and weight, if corrected for biases associated with socio-demographic characteristics of the survey respondents, can be used to provide accurate estimates of the proportion of overweight members of the population, but they still underestimate the proportion of obese population members. On the other hand, adjusted self-report measures do provide accurate estimates of health risks associated with variations in BMI, particularly when using parametric prediction models. Still, given the costs of data collection and the ability to reduce, even though not eliminate, systematic biases through adjustments using a few demographic variables, BMI values based on self-reported height and weight remain an important tool for population-based estimates of the health risks involved in obesity.

## Conclusion

BMI values based on self-reported height and weight, if corrected for biases associated with socio-demographic characteristics of the survey respondents, can be used to provide accurate estimates of the proportion of overweight members of the population, but they still underestimate the proportion of obese population members. On the other hand, adjusted self-report measures do provide accurate estimates of health risks associated with variations in BMI, particularly when using parametric prediction models.

## Competing interests

The authors declare that they have no competing interests.

## Authors' contributions

MS contributed most of the writing and the analysis. CAS assisted with the creation of the analytic file, analysis and the editorial review/writing.

## Authors' Information

MS is the 2008-2009 Academy Health Senior Service Fellow at the National Center for Health Statistics and Professor, Health Services Research, College of Nursing, Michigan State University

CAS has the position of Health Statistician in the Division for Health Interview Statistics, Centers for Disease Control, National Center for Health Statistics, Hyattsville, MD.

## Pre-publication history

The pre-publication history for this paper can be accessed here:

http://www.biomedcentral.com/1471-2458/9/421/prepub
